# Cancer-associated retinopathy secondary to gallbladder carcinoma

**DOI:** 10.3205/oc000243

**Published:** 2024-09-23

**Authors:** Karel Goyvaerts, Tanja Coeckelbergh, Pieter-Paul Schauwvlieghe, Michel van Lint

**Affiliations:** 1 Antwerp University Hospital, Department of Ophthalmology, Edegem, Belgium; 2 ZNA Middelheim Hospital Antwerp, Department of Ophthalmology, Antwerp, Belgium; 3 Brussels University Hospital, Department of Ophthalmology, Jette, Belgium

**Keywords:** cancer-associated retinopathy, gallbladder cancer, neuroendocrine tumor

## Abstract

**Objective:**

To present a rare case of cancer-associated retinopathy secondary to gallbladder carcinoma.

**Methods:**

Retrospective case report. Drugs used in case report: methylprednisolone (Medrol), CAS number: 83-43-2, producer: Pfizer; carboplatin, CAS number: 41575-94-4, producer: Accor; etoposide, CAS number: 33419-42-0, producer: Teva; methotrexate (Ledertrexate), CAS number: 59-05-2, producer: Pfizer.

**Results:**

A 57-year-old Moroccan man was referred with bilateral progressive vision loss in the last 4 months. At presentation, best corrected visual acuity (BCVA) was counting fingers for the right eye and 20/500 for the left eye. Examination demonstrated signs of vitritis, an electronegative full-field electroretinography (FF-ERG), ocular coherence tomography (OCT) abnormalities and multiple hyperautofluorescent round lesions on fundus autofluorescence imaging (FAF). The diagnosis of cancer-associated retinopathy (CAR) was considered, thus a positron emission tomography-computed tomography (PET-CT) was performed and revealed the presence of a metastasized gallbladder carcinoma. Additional fluorescence in situ hybridization (FISH) showed seropositivity for anti-retinal autoantibodies. High-dose corticosteroids together with anti-tumoral medication (carboplatin-etoposide) gradually improved the BCVA to 20/66 for the right eye and 20/20 for the left eye.

**Conclusions:**

Consider the diagnosis of CAR in patients with progressive concentric visual field loss, uveitis and fundus abnormalities, especially if bilateral. If CAR is suspected, perform a full work-up: FF-ERG, OCT, and whole-body PET-CT. In the treatment of CAR, immunosuppressives are mostly used, combined with antitumoral therapy. However, in the long-term, progressive visual loss is expected in most cases.

## Introduction

Cancer-associated retinopathy (CAR) is a rare paraneoplastic disorder of the retina, caused by an underlying distant neoplasm. The symptoms and signs are unrelated to the direct physical effects of the tumor or its metastases. Molecular mimicry is the pathogenic mechanism driving CAR. The ophthalmic clinical symptoms and signs are secondary to antibodies against a primary tumor located away from the eye. These antibodies then cross-react with retinal antigens due to antigenic similarities to the primary tumor. This results in apoptosis of the photoreceptors and widespread retinal degeneration. It usually leads to a rapidly progressive visual deterioration [1], [2]. CAR can be found in a wide variety of human cancers. It is most commonly encountered in small cell lung carcinoma followed by gynecological cancers (breast, endometrial, cervical, ovarian) and hematological tumors. Other cancers that are less associated with CAR include: hepatocellular carcinoma, pancreatic cancer, colon cancer, thymoma, bladder cancer, prostate cancer and non-small cell lung carcinomas [1], [2]. Here, we present a rare case of CAR secondary to a gallbladder carcinoma with bilateral eye involvement. To the best of our knowledge, this is the second case report describing CAR in the presence of a gallbladder carcinoma (first publication was a Japanese publication in 2004, only abstract available) [3]. Other, non-CAR paraneoplastic disorders secondary to gallbladder carcinoma have been regularly described. Most of these paraneoplastic disorders are non-ocular (neurological, dermatological, rheumatological, endocrinological, gastro-intestinal, hematological), but some case reports of bilateral diffuse uveal melanocytic proliferation (BDUMP) secondary to a gallbladder tumor could be found. We discuss pathophysiology, diagnosis, treatment and prognosis. We compare our case report with earlier publications, which leads us to find some differences in disease course and treatment response. 

## Case description

**Figure 1:**
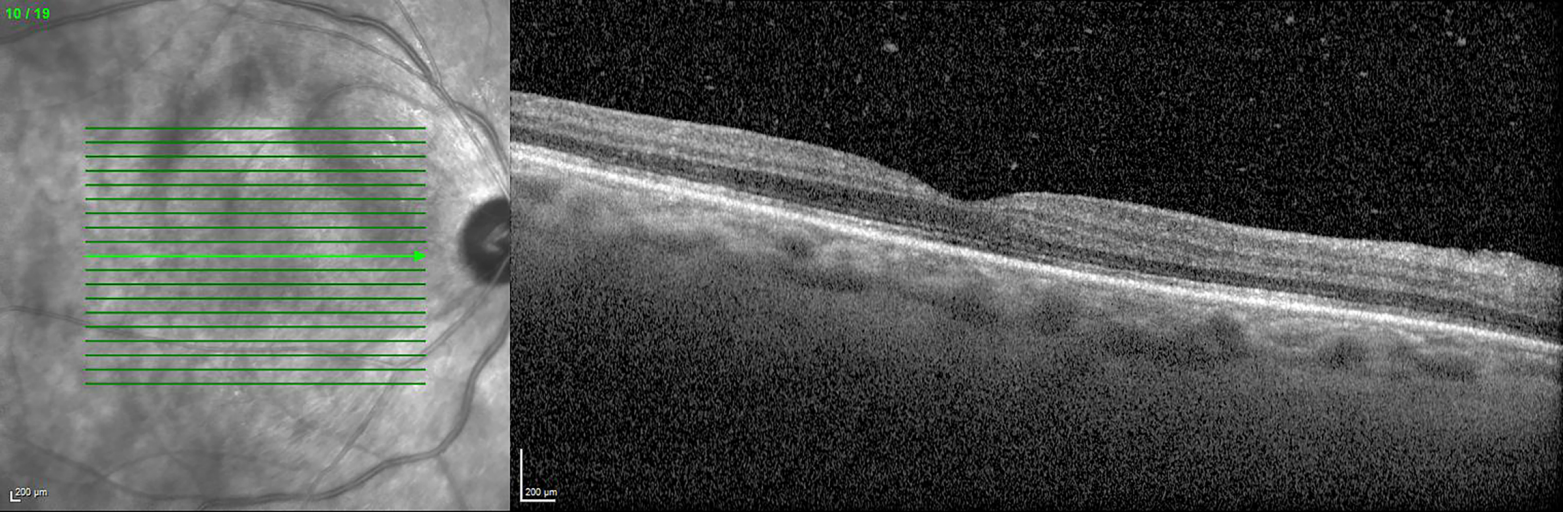
Optical coherence tomography (OCT): right eye shows vitreous opacities and slightly irregular ellipsoid zone (also visible in the left eye).

**Figure 2:**
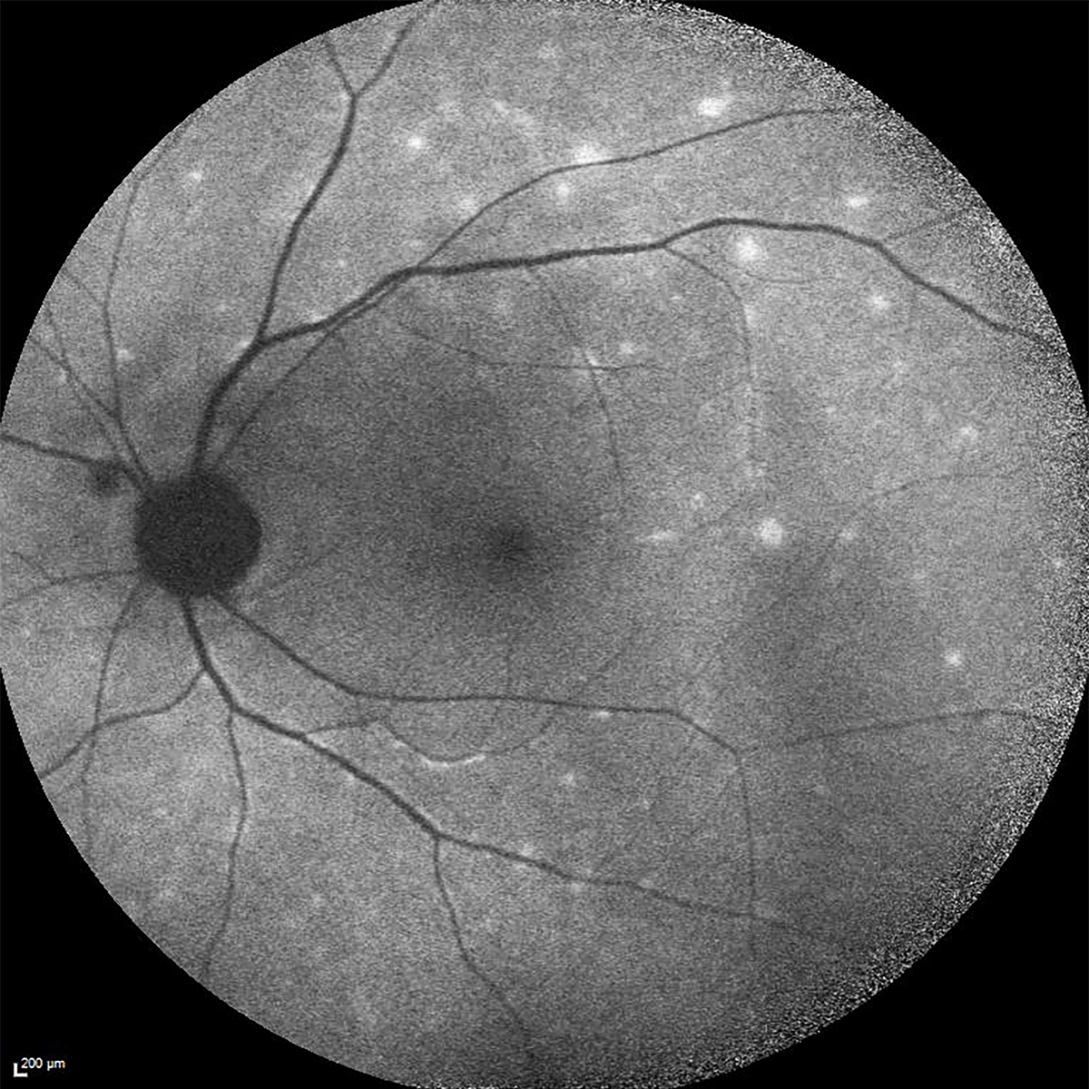
Fundus autofluorescence (FAF): hyper-autofluorescent lesions scattered in the posterior pole (left eye)

**Figure 3:**
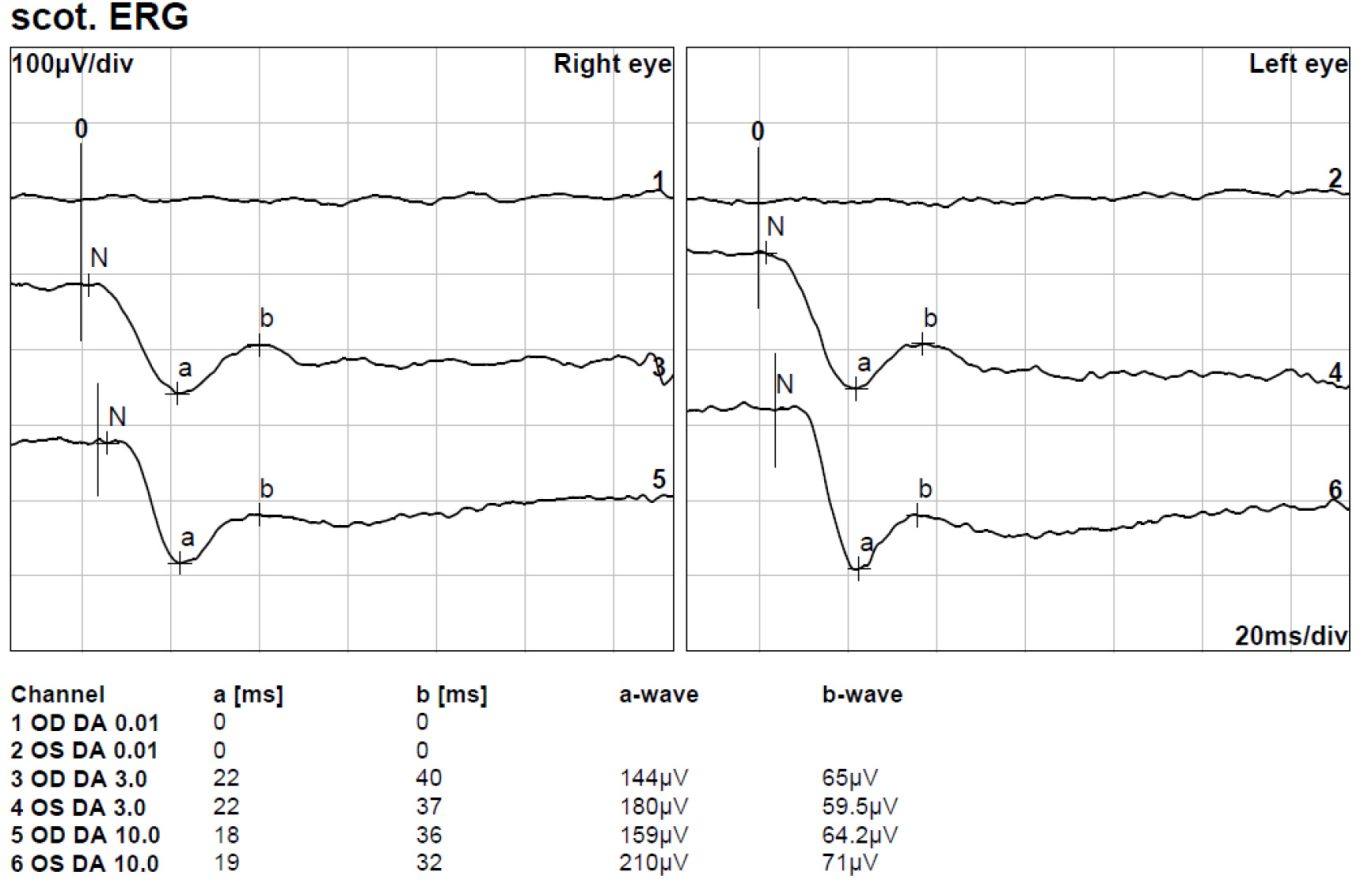
Full-field electroretinogram (FF-ERG) showing electronegative responses in both eyes. The amplitude of the b-waves stays well below baseline.

**Figure 4:**
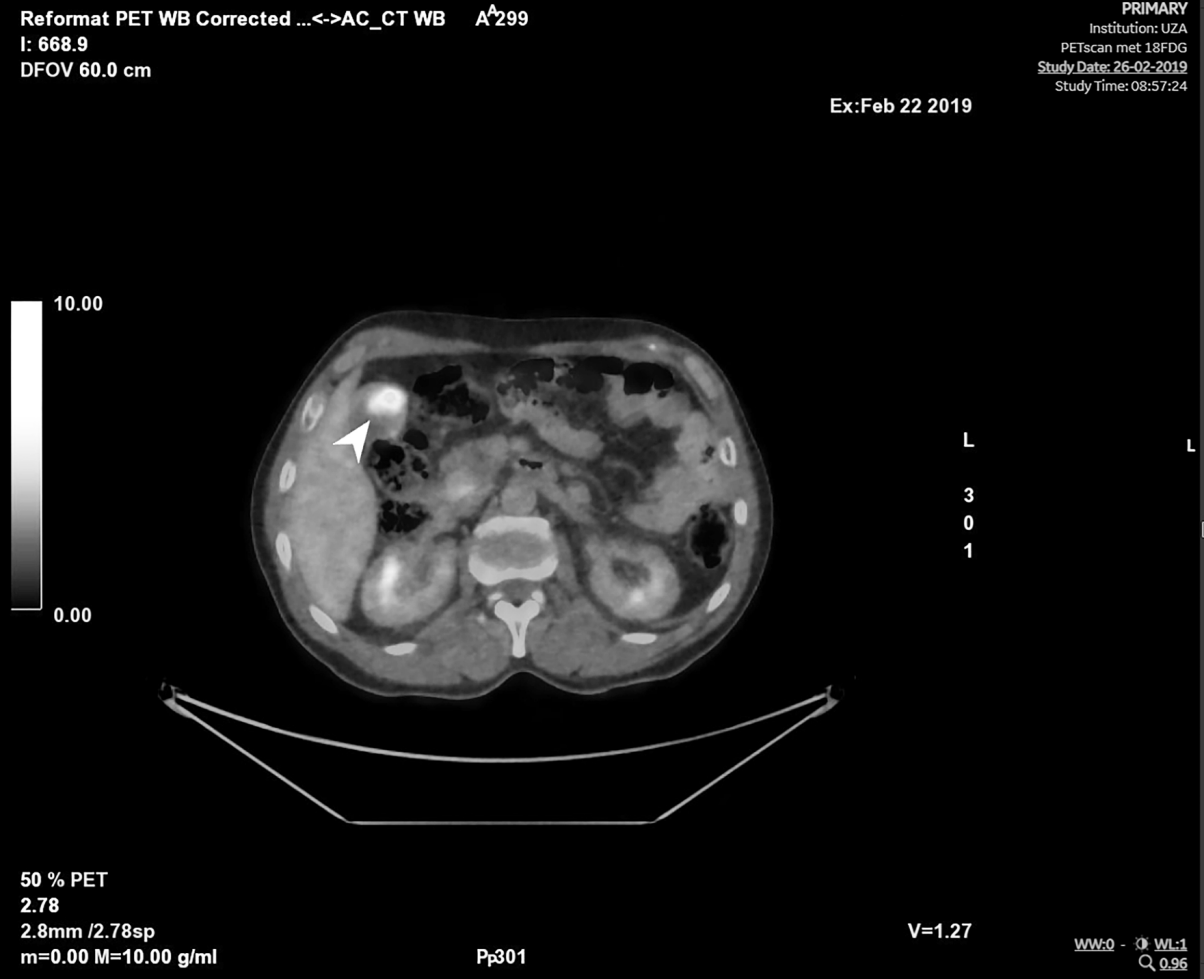
Positron emission tomography – computed tomography (PET-CT) shows gallbladder carcinoma, pathological lymphatic nodes near the liver hilus, and metastatic lesions in the pancreatic head and peritoneally at the right paracolic gutter.

**Figure 5:**
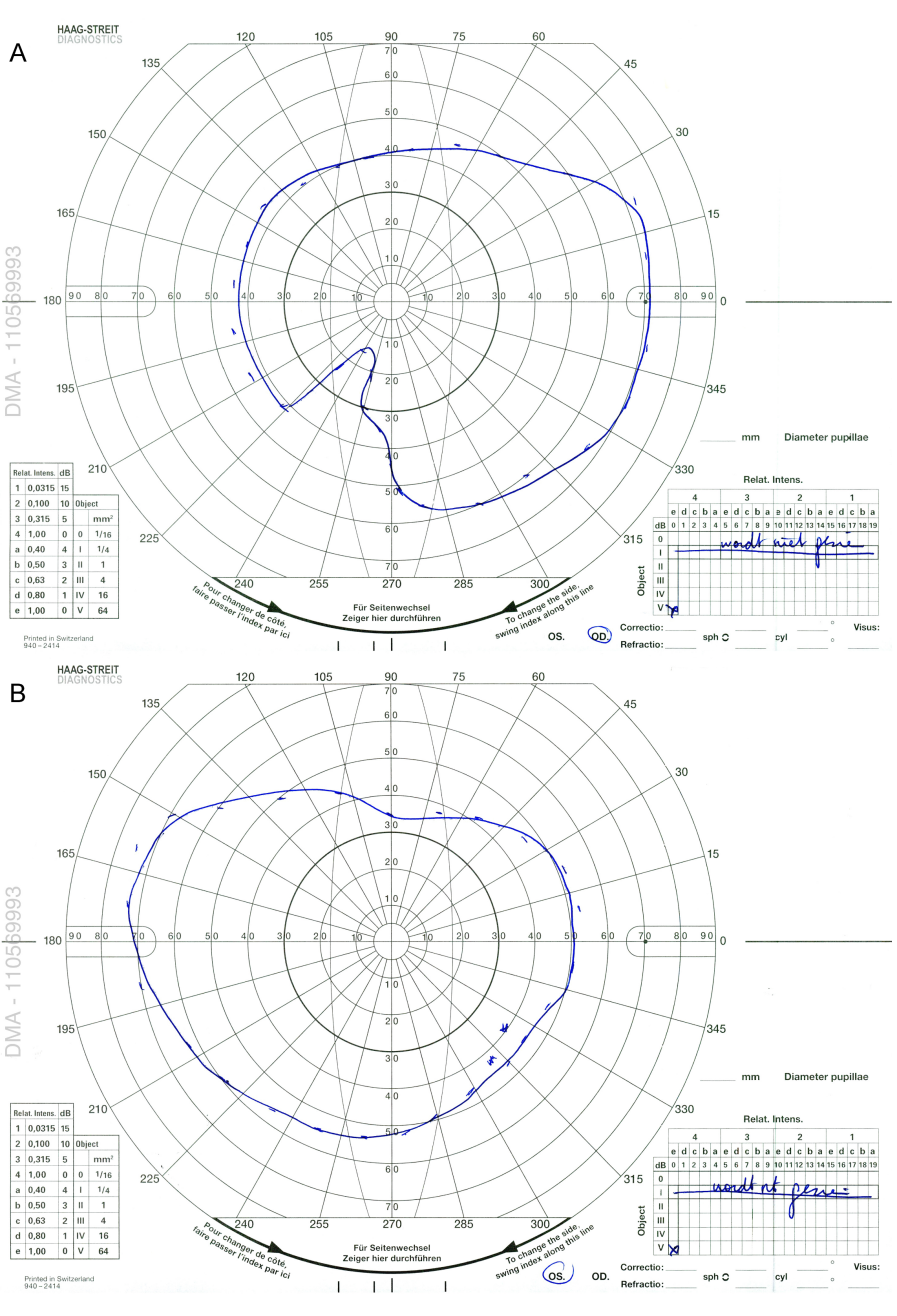
Goldmann visual field (VF) showed mild bilateral concentric narrowing of the visual fields: narrowing of the VF is more profound in the right eye (A), compared to the left eye (B). Size V4 stimuli were used.

**Figure 6:**
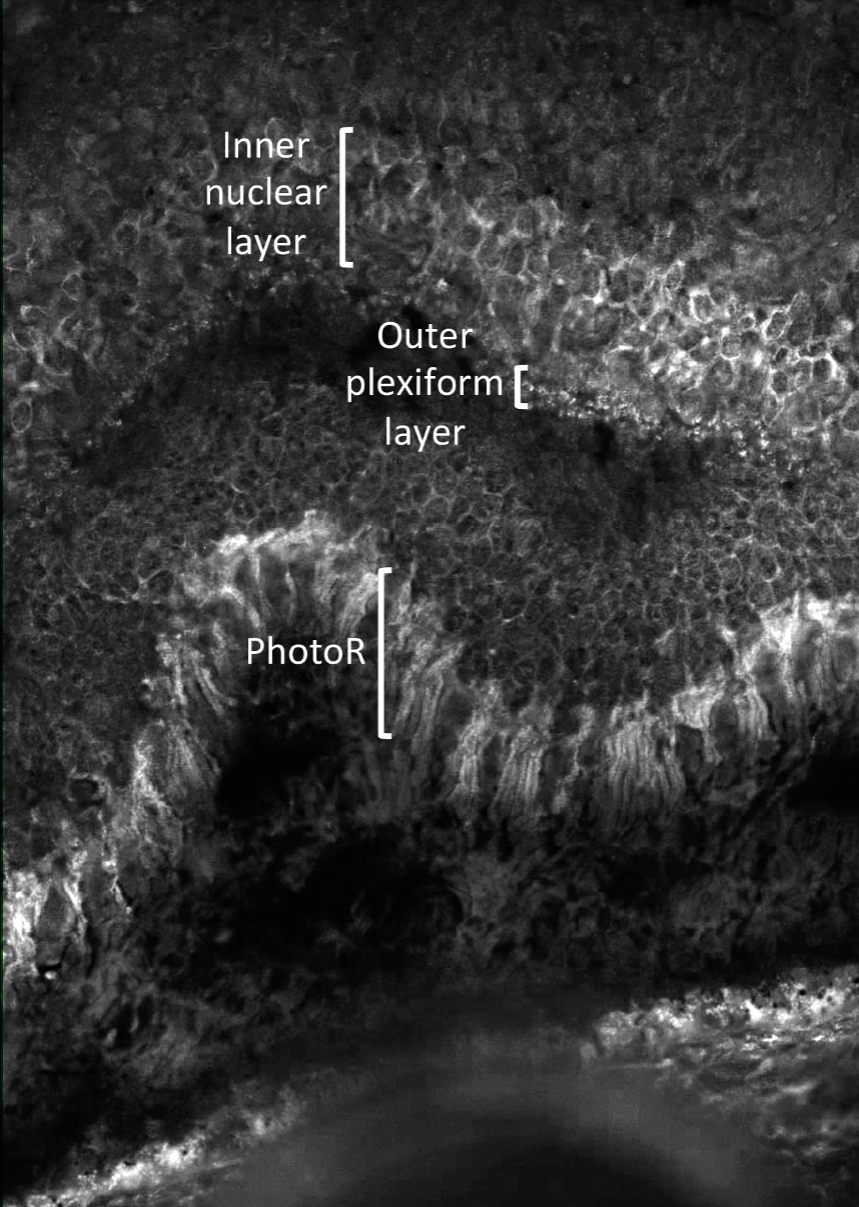
Indirect fluorescence in situ hybridization (FISH): primate retinal tissue containing antigens very similar to human retinal tissue was incubated with patient serum. After a wash off procedure, human IgG antibodies that bind fluorochrome were added to detect anti-retinal antibodies with fluorescence microscope. Anti-retinal autoantibodies against the inner nuclear layer (INL), outer plexiform layer (OPL) and photoreceptor layer (PhotoR) were detected.

A 57-year-old man from Morocco was referred 109 days after initial presentation for bilateral progressive visual loss and concentric narrowing of visual fields over a period of more than 4 months. Visual loss occurred simultaneously in both eyes, although more severely in the right eye. He experienced photo-aversion and a glare around objects. No previous personal or familial history of ocular complaints was found. Systemic history at presentation: hypovitaminoses D, smoking, surgical inguinal hernia repair. At presentation, best corrected visual acuity (BCVA) using the Early Treatment of Diabetic Retinopathy Study chart (ETDRS-chart) was counting fingers for the right eye and 20/500 for the left eye. Clinical examination demonstrated bilaterally a mild anterior chamber reaction, vitreous opacities (more pronounced in the right eye) and a retinoschisis-like aspect inferiorly with a profound bullous separation of the inner and outer retinal layers. Intra-ocular pressure (IOP) was normal, measuring 10 mmHg in the right and 11 mmHg in the left eye with Goldmann applanation tonometry. Optical coherence tomography (OCT) showed an irregular ellipsoid zone in both eyes with mild loss of the foveal depression in the right eye (Figure 1 (Fig. 1)). Multiple hyperautofluorescent round lesions were visible on fundus autofluorescence (FAF) in the posterior pole (Figure 2 (Fig. 2)). A differential diagnosis of paraneoplastic or non-paraneoplastic autoimmune retinopathy and lymphoma was considered. Vitreal biopsy was considered for laboratory investigation, but due to practical circumstances this was not performed. A full-field electroretinogram (FF-ERG) was performed, which yielded an electronegative response in both eyes (Figure 3 (Fig. 3)), possibly indicating loss of bipolar cell function and photoreceptor function. Clinical findings combined with the electronegative electroretinogram (ERG) made us suspect CAR. Whole-body positron emission tomography-computed tomography (PET-CT) was ordered by the ophthalmologists team and showed accumulation of fluor-18-deoxyglucose (18F-FDG) at the gallbladder, lymphatic nodes near the liver hilus, axial bone(-marrow), pancreatic head, and peritoneally at the right paracolic gutter (Figure 4 (Fig. 4)). These findings suggested a metastasized gallbladder carcinoma, supporting the clinical suspicion of CAR. Cerebral magnetic resonance imaging (MRI) did not reveal any metastatic lesions. A liver endoscopic lymph node puncture biopsy was performed and immunohistochemistry rendered a small cell neuro-endocrine tumor (NET) most likely, with Ki67 40%. Lymphoma was excluded. Because of the CAR-symptoms, a trial of high-dose oral corticosteroids (methylprednisolone 1 mg/kg/day; stepwise dose reduction after 40 days of treatment) was started by the ophthalmological team in consensus with the oncologists. Due to practical arrangements, the multidisciplinary oncological team started palliative chemotherapy with carboplatin-etoposide 10 days after the initiation of oral high-dose corticosteroids. Combined chemo- and oral corticoid-therapy led to the gradual improvement of visual acuity. 18 days after initiation of oral corticoids and 8 days after initiation of chemotherapy the vitreal haze had resolved. At this point attenuated retinal arterioles could be detected. After 4 months of treatment the ETDRS-chart BCVA comprised 20/66 for the right eye and 20/20 for the left eye. Goldmann visual fields became recordable for V4 light stimuli and showed only mild bilateral concentric narrowing of the visual fields (Figure 5 (Fig. 5)). From the oncological point of view, additional immunosuppressive agents were only deemed safe after 4 months into follow-up. To this end, methotrexate was started at a dose of 6x2.50 mg/week, along with folic acid at this moment in time (azathioprine was initially started, but had to be discontinued because it was not supported by the patient). Additional indirect fluorescence in situ hybridization (FISH) showed an autoimmune reaction against retinal antigens in the inner nuclear, outer plexiform and photoreceptor layers (Figure 6 (Fig. 6)). This indicated the presence of anti-retinal autoantibodies and confirmed the diagnosis of CAR. Anti-recoverin antibodies were negative, determination of other different subtypes of anti-retinal antibodies was not performed. Unfortunately, the patient died 9 months after diagnosis secondary to complications of his primary tumor.

## Discussion

The percentage of patients presenting with visual symptoms prior to cancer diagnosis in CAR, as seen in our patient, is still unclear as it varies between different studies, ranging from less than 4% (8 of the 209 patients) in the study of Adamus [4] to more than 50% as described by Naramala et al. [1]. The age of CAR patients ranges from 40 to 85 years (average 65 years) in the study of Adamus. In the same study, men were affected twice as often as women [4]. Our patient developed CAR secondary to a NET of the gallbladder. To the best of our knowledge, only one study has reported a CAR secondary to a gallbladder carcinoma; it was published in 2004 by Ban et al. in the Japanese Journal of Clinical Ophthalmology (only abstract available) [3]. McGrath et al. described a granulomatous anterior uveitis with vitritis in a patient with gallbladder carcinoma, but no diagnosis of CAR was described [5]. BDUMP is a paraneoplastic ocular syndrome that has been described before in patients with gallbladder cancer, but only a vast minority of BDUMP cases were secondary to gallbladder carcinomas [6]. PET-CT confirmation of a metastasized gallbladder carcinoma was only made 129 days after initial presentation with visual symptoms due to a delay in referral. Imaging for a primary tumor was ordered 20 days after initial presentation in our department after the FF-ERG (performed 16 days after initial check-up due to practical inconveniences) was electronegative, as this, together with the clinical signs and investigations described above, made the suspicion of a CAR very likely. An electronegative FF-ERG is common in melanoma-associated retinopathy (MAR), whereas FF-ERG in CAR usually affects first the a-wave and evolves rapidly to a flat/extinguished ERG [7]. This is not so in all cases as an electronegative FF-ERG has been described before in CAR patients in a case report by Goetgebuer et al. [8]. The fundus in CAR usually appears to be normal, but possible fundoscopic findings include vascular attenuation, optic disc pallor, and retinal pigment abnormalities. Inflammation can present as uveitis. OCT may demonstrate interruptions or loss of the ellipsoid layer, intraretinal cystic edema, or occasionally retinoschisis-like changes. Autoantibodies can be detected by immunohistochemistry (IHC), Western blot or an enzyme-linked immunosorbent assay (ELISA) [2], [3]. The most common retinal antigens are anti-recoverin, anti-α-enolase, anti-carbonic anhydrase II, heat shock cognate protein 70 (HSC70), anti-transducin-α, and anti-GADPH [1]. There is no consensus about the sensitivity and specificity of serum autoantibodies as different studies show different results. Although a higher incidence of antibody levels is seen in patients with CAR compared to control patients, it is still important to correlate clinical features and additional tests with the presence of anti-retinal antibodies to confirm the diagnosis [9]. Treatment options in CAR include the use of corticosteroids (systemic and/or topical), intravenous immunoglobulins (IVIG), rituximab, alemtuzumab, and plasmapheresis [1]. Treatment is mostly associated with mild to moderate improvement in visual function. Early use of immunosuppressive therapy seems to improve the chance of treatment response [2]. Despite initial visual improvement, long-term visual prognosis remains poor due to the persistence of anti-retinal antibodies that may persist for several years [10]. Antibody titers may be used for follow-up, as they correlate with disease activity [11]. Importantly, the primary tumor and its metastases also require appropriate treatment. In our patient, BCVA remained stable after 8 months into follow-up, which contrasts with some other case reports of CAR, reporting limited or temporary visual improvement under corticoid therapy. Difference in therapy response could be due to the heterogeneity of autoantibodies. Unfortunately, as shown in this case, the ophthalmic treatment response does not imply longer survival. This still depends on the underlying cancer, its staging at diagnosis, and treatment options for the primary cancer and its metastases [2], [3].

## Conclusion

In conclusion, we report a rare case of cancer-associated retinopathy secondary to a metastatic NET of the gallbladder. The ophthalmic signs and symptoms led to the diagnosis of the primary tumor. It is important to consider CAR in cases of unexplained progressive visual field loss, uveitis, and fundus abnormalities. Photo-aversion, prolonged glare after light exposure, decreased colour perception, and central scotomas may be part of the clinical spectrum. Heterogeneity of autoantibodies may explain the variation and complexity of clinical symptoms between patients [11]. If CAR is suspected, it is recommended to perform a full work-up, including full-field ERG, OCT, and whole-body PET-CT. Immunosuppressive and anti-tumoral therapy should be started without unnecessary delay and in cooperation with the treating oncologist so as not to negatively affect life expectancy.

## Notes

### Patient consent

Patient consent to publish this case report could not be obtained due to the patient’s decease. This report does not contain any personal information that could lead to the identification of the patient, however.

### Competing interests

The authors declare that they have no competing interests.
